# Data-Driven Healthy China Pathway: Evolution, Framework, and Global Implications of National Digital Health Strategic Planning

**DOI:** 10.2196/89099

**Published:** 2026-07-20

**Authors:** Jialin Liu, Siru Liu

**Affiliations:** 1 Department of Medical Informatics, West China Hospital, Sichuan University, Chengdu, Sichuan, China; 2 Department of Otolaryngology-Head and Neck Surgery, West China Hospital, Sichuan University, Chengdu, Sichuan, China; 3 Department of Biomedical Informatics, Vanderbilt University Medical Center, 2525 West End Avenue Suite 1475, Nashville, TN, 37203, United States, 1 (615) 936-6867

**Keywords:** digital health, health policy, data governance, continuous planning, Healthy China 2030, health information systems, artificial intelligence, AI

## Abstract

Amid accelerating digital health transformation, China has developed a centrally coordinated data-driven healthy China pathway. This viewpoint conceptualizes this pathway as a hybrid continuous planning framework (HCPF) that links long-term strategic direction with short-cycle tactical adaptation. China’s strategy has progressed through 3 overlapping periods: infrastructure-oriented periodic planning (2015-2018), emergency-driven digital acceleration (2019-2021), and institutionalized continuous planning (2022-2025). The HCPF emerged from cumulative policy layering: Healthy China 2030 set long-range goals, 5-year plans translated these goals into programmatic priorities, annual adjustments enabled recalibration, and emergency response mechanisms and technical iterations supported adaptive implementation. Official reports indicate that China approved more than 2700 internet hospitals by 2024, and by December 2025, a total of 9073 secondary and tertiary public hospitals and 503,000 primary-level public hospitals and grassroots medical and health institutions were connected to regional platforms. The HCPF has supported large-scale data governance, interdepartmental coordination, pilot-based implementation, platform connectivity, and rapid pandemic response. However, it also raises risks related to data privacy, regional disparities, performative compliance, and the burden of agility on grassroots institutions. The transferable lesson is not direct replication of China’s model but the design of temporally layered strategies that combine central coordination local adaptation, data-informed feedback; and safeguards for privacy, equity, and frontline capacity. Digital health incentives should prioritize meaningful clinical value and system connectivity rather than infrastructure adoption alone.

## Introduction

China’s digital health strategy has evolved over the past decade from a set of sectoral initiatives into an integrated national program for Healthy China 2030 [1]. This transformation has unfolded within one of the world’s largest and most heterogeneous health systems. By the end of 2024, China had more than 1.09 million medical and health institutions, including 38,710 hospitals and roughly 570,000 village clinics, supported by more than 13 million health technical personnel [2]. At the same time, digital infrastructure has expanded rapidly: China approved more than 2700 internet hospitals by 2024, and by December 2025, a total of 9073 secondary and tertiary public hospitals and 503,000 primary-level public hospitals and grassroots medical and health institutions were connected to regional health information platforms [3,4]. These figures reflect platform connectivity rather than the total number of institutions, but they illustrate the central governance challenge: national digital health planning must coordinate system scale, institutional heterogeneity, and uneven digital maturity.

This policy trajectory began with the 2015 Guiding Opinions on Actively Promoting the “Internet Plus” Action Plan by the State Council, which framed the internet and data as critical infrastructure for economic and social transformation [5]. It was subsequently anchored by 3 landmark frameworks: the Healthy China 2030 Planning Outline (2016) [1]; the Opinions on Promoting the Development of “Internet Plus Medical Health” [6]; and the 14th Five-Year Plan for National Health together with the 14th Five-Year Plan for National Health Informatization (2021-2025), both issued in 2022 [7,8]. These policy instruments have helped produce a data-driven healthy China pathway that combines centralized strategic direction, large-scale data infrastructure, national implementation mechanisms, and strong resource mobilization capacity. However, implementation capacity remains uneven across regions, with more mature platforms concentrated in eastern and urban pilot areas and persistent gaps in many western, rural, and grassroots institutions [9-14]. This unevenness motivates the hybrid continuous planning framework (HCPF) lens, which explains how national digital health planning seeks not only to expand infrastructure but also to coordinate heterogeneous institutional capacities through national standards, platform connectivity, pilot-based scaling, and iterative policy adjustment.

China’s digital health strategy can be summarized in 3 overlapping phases. From 2015 to 2018, an infrastructure-oriented periodic planning phase, governed mainly through 5-year plans and key performance indicators, emphasized electronic medical record (EMR) maturity evaluation in tertiary hospitals and the development of national and regional health big data centers. From 2019 to 2021, COVID-19 accelerated health code systems, internet hospitals, and remote consultations from pilots to mainstream service delivery [15-17], shifting implementation temporarily from multiyear cycles to week- or month-level iteration under emergency governance. Since 2022, institutionalized continuous planning has linked long-range visions, 5-year plans, annual rolling adjustments, and agile emergency response or technical iterations through national health information standards and platform interconnection goals [8,18,19]. Prior studies have examined China’s digital health policies, including their alignment with global digital health frameworks and their “whole-of-society” governance features [20,21], but less attention has been paid to the temporal and adaptive logic of this planning process. This viewpoint is informed by an interpretive synthesis of national policy documents, policy implementation documents, published digital health studies, legal and regulatory materials, and documented implementation experiences in China, focusing on strategic planning, health information infrastructure, data governance, institutional implementation mechanisms, regional disparities, and governance risks.

Despite substantial policy and infrastructure achievements, the planning logic underlying China’s data-driven healthy China pathway remains insufficiently conceptualized, particularly how long-term national goals are aligned with rapidly changing digital technologies, unequal implementation capacity, and crisis demands. This viewpoint, therefore, advances three linked arguments: (1) China’s digital health strategic planning has moved from relatively static periodic cycles toward a hybrid continuous planning paradigm; (2) the HCPF links decadal visions, 5-year plans, annual rolling adjustments, emergency response mechanisms, and continuous technical iteration within one governance architecture; and (3) the framework’s practical operation depends on central coordination, pilot-driven investment, public-private-academic collaboration, and performance-linked incentives while requiring safeguards against data governance risks, regional disparities, performative compliance, and the burden of agility. The intended audience includes digital health policymakers, national and regional health system leaders, hospital executives, health informatics leaders, and researchers in digital health governance and health policy. This manuscript is organized accordingly: it traces the evolution of China’s digital health planning; explicates the HCPF as a governance architecture; and examines its implementation mechanisms, governance risks, and cautious global implications.

## The Chinese HCPF

### Conceptual Positioning and Originality

The HCPF draws on adaptive governance, adaptive policymaking, digital health strategy, and learning health systems but reorients these ideas toward national digital health strategic planning [22-25]. Its originality lies in explaining how long-term direction and short-cycle adaptation can be connected through a temporally layered planning architecture. The framework also highlights how top-down mobilization, bottom-up data feedback, and regional disparities together affect the quality and representativeness of policy recalibration. It is therefore best understood not as a new universal governance theory but as a digital health–specific lens for studying the shift from static planning toward adaptive governance in a large, heterogeneous health system.

### Framework Architecture and Operational Logic

**Figure 1.**
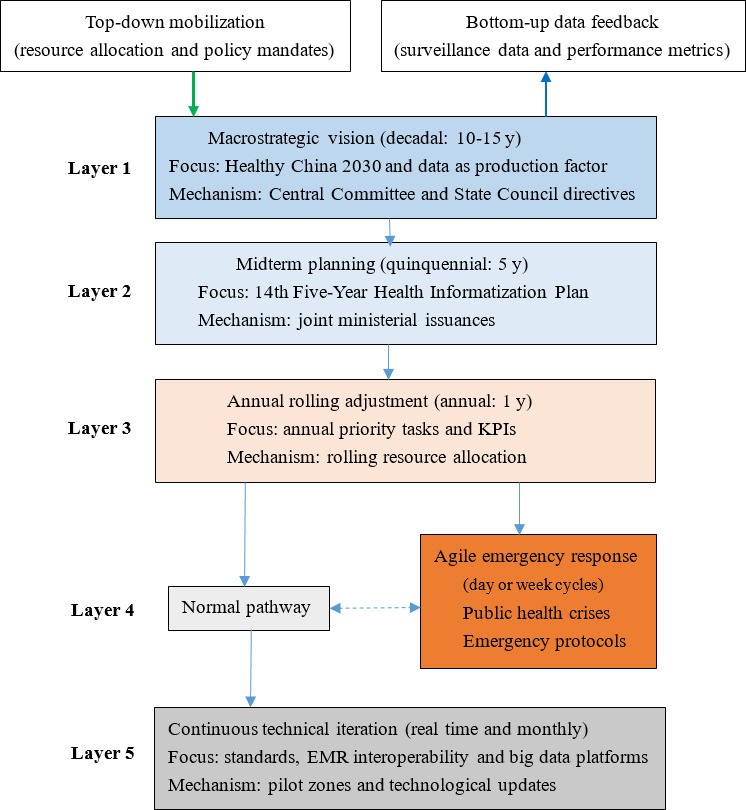
Hybrid continuous planning framework (HCPF) governing the data-driven healthy China pathway. This figure illustrates the HCPF as a temporally layered governance architecture for China’s data-driven healthy China pathway. Long-term national strategies and 5-year plans provide strategic direction; annual rolling priorities support recalibration; emergency mechanisms enable rapid response; and continuous technical iteration periodically updates standards, platforms, and implementation tools. Two linked flows sustain this architecture: top-down mobilization through policy mandates, resource allocation, and performance incentives, and bottom-up feedback through surveillance data, platform metrics, pilot evaluations, and institutional reporting. EMR: electronic medical record; KPI: key performance indicator.

The data-driven healthy China pathway can be conceptualized through the HCPF, which integrates long-range strategic direction with shorter-cycle policy, emergency, and technical adjustments. Unlike linear planning models, the HCPF operates as a temporally layered governance architecture that supports vertical alignment while allowing for adaptation to technological and epidemiological uncertainty. The architecture contains 5 interdependent tiers organized into strategic anchors and dynamic engines (Figure 1). Table 1 traces how these tiers emerged across China’s 3 planning phases and summarizes their implications for the framework.

**Table 1.** Evolution of China’s national digital health strategies and implications for the hybrid continuous planning framework (HCPF)^a^.

Planning period	Dominant planning logic	Key policy instruments	Implementation outcomes and regional implications	Implications for the HCPF
2015-2018: infrastructure-oriented periodic planning	Five-year planning and top-down infrastructure construction	“Internet Plus” action plan, Healthy China 2030 Planning Outline, Opinions on Promoting the Development of “Internet Plus Medical Health,” and early health big data and EMR^b^ maturity initiatives	National and regional health big data centers, EMR maturity evaluation, and early internet-based medical services expanded. Tertiary hospitals and eastern pilot areas accumulated stronger digital infrastructure, whereas many grassroots and western institutions remained reliant on fragmented local systems.	Established the HCPF’s strategic anchor by linking digital health to national development goals, infrastructure investment, and performance-based implementation
2019-2021: emergency-driven digital acceleration	Crisis response, rapid mobilization, and short-cycle policy iteration	COVID-19 emergency governance, health code systems, and rapid expansion of internet hospitals and remote consultation	Health codes, online consultation, internet hospitals, and telemedicine expanded rapidly from pilots to large-scale service delivery. High-resource regions scaled digital tools more quickly, whereas low-resource regions faced weaker connectivity, limited informatics capacity, and heavier reporting burden.	Added the HCPF’s emergency agility layer by showing how planning could shift from multiyear cycles to week- or month-level adaptation during public health crises
2022-2025: institutionalized continuous planning	Integration of long-range vision, 5-year plans, annual rolling adjustment, and technical iteration	14th Five-Year Plan for National Health, 14th Five-Year Plan for National Health Informatization, and national health information standards and platform interconnection goals	EHR^c^ development, public institution platform connectivity, functional health codes, internal hospital information sharing, and nationwide sharing of core information across tertiary hospitals advanced. Persistent gaps remained in interoperability, vendor dependence, data quality, and institutional capacity to absorb frequent mandates.	Formalized the HCPF as a temporally layered architecture combining long-range vision, rolling adjustment, technical iteration, and bottom-up data feedback

a Across the 3 phases, planning logic shifted from infrastructure-oriented periodic construction toward institutionalized continuous planning, whereas gaps between digitally mature and less mature regions persisted and shaped each phase’s contribution to the HCPF. “EMR” and “EHR” are used deliberately and are not interchangeable: earlier phases emphasized electronic medical record maturity within individual hospitals, whereas the most recent phase emphasized resident-level electronic health record development and cross-institutional data sharing. The table reflects the dominant focus of each phase rather than rigid boundaries as planning logics overlap across periods.

b EMR: electronic medical record.

c EHR: electronic health record.

### The Strategic Anchors: Defining Direction and Pace

At the apex, the long-term strategic vision (10- to 15-year horizon) defines enduring goals. Blueprints such as Healthy China 2030, formulated by the Central Committee and State Council, set broad priorities such as population health equity, whereas later directives elevated data, including health data, as a factor of production [1,26]. This layer sets strategic direction rather than concrete implementation pathways. These visions are translated into programmatic commitments through midterm planning (5-year horizon), exemplified by the 14th Five-Year Plan for National Health Informatization [18]. Through joint central agency issuances, this layer identifies priority domains, such as regional big data centers and telemedicine expansion, and sets measurable milestones [8]. Annual rolling adjustments and periodic midterm evaluations then moderate the rigidity of 5-year planning. This bridging layer translates midterm goals into actionable annual tasks, including interoperability targets and pilots for diagnosis-related group (DRG) and diagnosis-intervention packet (DIP) payment reform [27], allowing priorities to be resequenced in response to budget constraints, implementation feedback, and emerging technologies.

### The Dynamic Engines: Ensuring Resilience and Evolution

The framework’s adaptive capacity resides in its lower tiers. The agile emergency response layer operates on day-to-week cycles and enables rapid cross-agency mobilization during public health crises. During the COVID-19 pandemic, for example, health code risk classification rules were iteratively updated in response to changing case patterns and mobility needs [28]. The base layer is continuous technical iteration: foundational standards and tools are refined without waiting for the next formal planning cycle, often beginning as regional pilots before national scaling. Examples include the gradual tightening of EMR grading criteria and the staged rollout of DRG and DIP grouping standards. These standards function as payment and management tools that classify clinical cases and support standardized reimbursement and hospital performance management.

### Operational Dynamics: The Dual-Directional Feedback Loop

Functionally, the HCPF operates through dual-directional flows. Top-down mobilization promulgates mandates and allocates resources across administrative levels. Bottom-up data intelligence, derived from surveillance data, performance assessments, and pilot evaluations, informs strategic recalibration. In practice, this feedback loop operates through a tiered data governance pathway. National authorities define policy priorities, technical standards, data elements, security requirements, and performance indicators. Provincial platforms translate these requirements into regional health information architectures. Municipal and county systems aggregate data from hospitals, primary care institutions, public health agencies, and payment systems. Local institutions then generate, validate, and upload clinical, administrative, and public health data. This feedback loop connects top-level design with frontline implementation and allows the national strategy to adjust based on empirical signals [29,30]. However, the quality of this loop depends on local digital maturity. In digitally mature regions, data governance can be supported by integrated regional platforms, interoperability interfaces, hospital performance dashboards, and pilot-based feedback mechanisms. In less digitally mature regions, implementation may depend on minimum dataset requirements, provincial cloud-based services, targeted fiscal support, technical assistance from higher-level hospitals, and staged adoption of electronic health records and interoperability standards. The result is a hybrid planning model in which long-term and 5-year plans provide stability, annual and technical iterations enable incremental adjustment, and emergency mechanisms permit rapid reconfiguration. Its core contribution is temporal integration: aligning decisions made across decadal, 5-year, annual, and subannual time scales. This alignment helps explain how China has pursued long-term digital health goals while adapting to technological change and public health shocks.

## Implementation Mechanisms Enabling Large-Scale Digital Health Transformation

### Robust Central Coordination and Institutional Synergy

China uses a whole-of-government approach. The National Health Commission serves as the lead health authority, whereas agencies responsible for cybersecurity, data regulation, and infrastructure planning, including the Cyberspace Administration of China and the National Development and Reform Commission, contribute to coordination. The National Data Administration further reflects the strategic elevation of data governance and data resource development [31]. This cross-agency structure can reduce fragmentation by aligning standards, infrastructure investment, data governance, and hospital performance requirements across different levels of government while linking these efforts to broader national digital economy strategies.

### Data-Centric, Pilot-Driven Investment Prioritization

China has concentrated fiscal and regulatory support on designated pilot regions rather than pursuing uniform national rollouts [32]. Central authorities use fiscal transfers and “new infrastructure” (*xin jijian*) funding to support national and provincial health care big data centers, digital health pilot cities, and demonstration zones [33]. These sites receive targeted funding, policy support, and technical assistance for interoperability infrastructure, cloud platforms, and analytics capabilities [7]. They function as experimentation fields for EMR adoption, regional interoperability, DRG and DIP payment models, and data governance and implementation mechanisms [34]. This pilot-based strategy allows digital maturity models and governance arrangements to be tested in more digitally mature settings before wider diffusion.

### Public-Private-Academic Innovation Ecosystems

The Chinese model combines public governance, private technological capability, and academic validation. Public authorities retain responsibility for data governance and safety regulation while drawing on the technical capabilities and consumer infrastructure of platform companies [35,36]. Academic medical centers and research universities provide clinical datasets and testing environments for refining AI algorithms and medical standards before wider scaling [37]. Companies such as Alibaba Health and Tencent Medipedia, together with medical AI developers, collaborate with hospitals, academic medical centers, and research institutes to develop telemedicine platforms, AI-assisted diagnostic tools, and health information portals [38,39]. Integrated services in WeChat, Alipay, and similar platforms provide a consumer-facing digital front door for appointment scheduling, payment, telehealth, and health information [40]. This configuration blends state steering with market experimentation while reducing the need for the health system to build all consumer-grade infrastructure independently.

### High-Stakes Performance Incentives

A major driver is the integration of digital metrics into the National Tertiary Public Hospital Performance Appraisal (colloquially known as the Guokao), which assesses China’s tertiary public hospitals. Indicators such as EMR application-level grading, interoperability maturity, and smart hospital ratings are weighted components of performance assessment [41]. Because Guokao results can influence accreditation, resource allocation, and leadership appraisal, they function as a significant policy lever for digital transformation. By tying EMR maturity and data quality to hospital performance, the state incentivizes hospital executives to invest in digital infrastructure, data governance, and clinical adoption, turning digitalization from a technical support function into an institutional priority linked to competitiveness and leadership accountability [42].

## Governance Risks and Critical Reflections

### Data Governance, Privacy, and Public Trust

China’s large-scale deployment of health codes (*jiankang ma*), itinerary codes, and nucleic acid testing registries during the COVID-19 pandemic response constituted one of the most extensive real-time collections of personal health and mobility data in recent history. Although these systems contributed substantially to pandemic control, they also exposed systemic governance challenges regarding data privacy and data protection [43]. The Personal Information Protection Law (PIPL; 2021) treats medical and health information as sensitive personal information and requires specific purpose, sufficient necessity, strict protective measures, and separate consent for its processing (PIPL Articles 28-29) [44]. The Data Security Law (2021) further establishes a broader framework for data classification, risk management, and data security obligations [45]. Despite these legal frameworks, local implementations in some jurisdictions appear to have exceeded statutory or proportionality limits. Many municipal platforms collect granular data beyond epidemiological necessity, whereas the algorithmic rules governing code assignment and reversion remain opaque [16,46]. Moreover, prior analyses have raised concerns about function creep, in which health code data could be repurposed for objectives such as social stability maintenance or public security rather than disease control alone [47]. Rebuilding legitimacy, therefore, requires more than reliance on existing framework legislation. Targeted reforms could include (1) dedicated regulations on personal information processing during public health emergencies, including clear retention limits such as mandatory deletion of identifiable data after the emergency period absent a separate lawful basis [46]; (2) an independent, cross-ministerial health data oversight mechanism with authority to audit algorithms and data flows; and (3) lower barriers to individual redress, including clarification of the public interest litigation threshold associated with PIPL Article 70 [48], so that affected individuals can more readily challenge erroneous code assignments or data misuse.

### Regional Disparities and the Digital Divide

A major vulnerability of the pathway is the persistent data divide between digitally mature eastern or urban pilot zones and western, rural, or grassroots settings [9-11]. The risk is not only unequal infrastructure but also feedback bias: if planning data come mainly from high-resource regions, national strategies and AI models may underrepresent rural health needs. Usable feedback depends on data completeness, coding consistency, workflow integration, local informatics capacity, and sustained data quality work. In high-resource settings, these functions are supported by mature hospital information systems, regional platforms, dashboards, and automated quality control mechanisms. In low-resource settings, data may be incomplete, delayed, manually entered, or difficult to integrate across vendor-specific systems, weakening their influence on planning and policy recalibration.

To avoid a 2-speed digital transformation, governance must move from passive standardization to active redistribution [12,13]. Priority measures could include (1) earmarked central fiscal transfers for last-mile connectivity and basic clinical decision support in county-level facilities; (2) provincial cloud-native architectures that provide grassroots institutions with standardized software-as-a-service applications, that is, centrally hosted software delivered through the cloud rather than installed and maintained locally without heavy local maintenance costs; (3) simplified reporting requirements combined with stronger data quality auditing so that grassroots institutions are not overwhelmed by redundant data entry mandates; (4) formal frontline feedback channels before national scaling of new standards; and (5) “east-west” informatics twinning programs linking digitally mature hospitals with lower-resource institutions. These measures would help ensure that national data infrastructure reflects the epidemiological reality of the entire population, not only the urban core [12,14].

### Sustainability of Incentive Structures: The Trap of Performative Compliance

The HCPF’s performance-linked incentives mobilize investment but can also reinforce “heavy construction, light application” (*zhong jianshe, qing yingyong*) [49-51]. Under Guokao pressure, hospitals may prioritize EMR functional level 5 or 6 certification to improve rankings [52]. This can encourage documentation templates or structured data fields designed to satisfy scoring algorithms rather than improve care coordination. The result may be technically compliant but clinically burdensome systems that increase redundant data entry and reduce usability [53,54]. Sustainable incentive design should therefore shift from static technical specifications, such as interface counts, toward dynamic clinical value, such as effects on clinician workload, care coordination, and patient outcomes.

Reforms could include (1) outcome-based metrics, such as reductions in readmission rates tracked via integrated EMR–public health platforms assigned substantial weight to counterbalance process-oriented indicators, with penalties for unverifiable data; (2) hybrid evaluations combining automated data logic verification with unannounced on-site audits (*feixing jiancha*) to detect gaming behaviors and ensure consistency between reported data and actual clinical workflows [41]; and (3) incentives to mobilize private investment, such as tax rebates for enterprise-hospital collaborations, to supplement government subsidies and reduce overreliance on direct fiscal levers. Such adjustments, consistent with the value-based evolution of the US Meaningful Use program [55], would better align the HCPF with equitable, outcome-driven digital transformation.

### The Burden of Agility: Structural Asymmetry and Reform Fatigue

A critical challenge within China’s data-driven pathway is the structural asymmetry between the central government’s rapid policy iteration and the limited absorptive capacity of frontline institutions. The convergence of high-frequency technical mandates from multiple ministries, including the National Health Commission, Cyberspace Administration of China, and Ministry of Industry and Information Technology, has created a fragmented compliance landscape in which grassroots providers must navigate overlapping interface standards and redundant reporting requirements [56]. This administrative burden can contribute to reform fatigue and digital formalism, leading local implementers to prioritize technical compliance and data upload quotas over genuine workflow re-engineering [57-59]. Although top-down performance incentives have accelerated infrastructure deployment [41,42], they may also decouple data generation from clinical utility, particularly in resource-constrained subcounty settings [59-61]. Resolving this asymmetry requires shifting from rapid mobilization to sustainable integration, specifically by introducing stability windows for technical standards and granting frontline institutions greater discretion to adapt noncore functions to local needs.

## Conclusions

China’s HCPF illustrates one approach to governing digital health transformation, not a universal model for direct replication. Its value lies in showing how long-term policy direction, periodic planning, annual recalibration, emergency response, and continuous technical iteration can be connected within a single planning architecture. This logic aligns with the World Health Organization *Global strategy on digital health 2020-2025* and the Global Initiative on Digital Health by treating digital health as a means to strengthen health systems rather than digitize isolated services. The transferable lessons are principles rather than blueprints: temporal layering, national-local coordination, data-informed feedback, accountable data governance, equity-oriented investment, and incentives linked to clinical value and system connectivity. The transferability of these principles will depend on local governance, financing, legal safeguards, and digital infrastructure. Where governance is decentralized, financing is fragmented, or data protection regimes are weak, countries should translate these principles into locally legitimate and feasible arrangements rather than reproduce China’s state-centric model.

## Abbreviations

DIP: diagnosis-intervention packet
DRG: diagnosis-related group
EMR: electronic medical record
HCPF: hybrid continuous planning framework
PIPL: Personal Information Protection Law
